# Kinematic Gait Adjustments to Virtual Environments on Different Surface Conditions: Do Treadmill and Over-Ground Walking Exhibit Different Adaptations to Passive Virtual Immersion?

**DOI:** 10.1155/2020/8901973

**Published:** 2020-12-19

**Authors:** Gonzalo Varas-Diaz, Shivani Paralkar, Shuaijie Wang, Tanvi Bhatt

**Affiliations:** ^1^Department of Physical Therapy, University of Illinois at Chicago, Chicago IL, USA; ^2^PhD Program in Rehabilitation Sciences, College of Applied Health Sciences, University of Illinois at Chicago, Chicago IL, USA

## Abstract

**Background:**

The aim of this study was to examine the kinematic gait adjustments performed in response to passive and photorealistic virtual reality environment (VRE) demands during over-ground and treadmill walking conditions and determine whether the surface presentation order affects the gait adjustments in response to different VREs.

**Methods:**

Twenty young participants divided into two groups performed two virtual reality (VR) walking protocols which included two different VREs (snowy and crowded conditions). Group A performed the VR over-ground protocol (four natural walking (NW), seven VR snowy, and seven VR crowded trials) followed by the VR treadmill protocol (four NW, one VR snowy, and one VR crowded trials); Group B performed the VR treadmill protocol (four NW, seven VR snowy, and seven VR crowded trials) followed by the VR over-ground protocol (four NW, one VR snowy, and one VR crowded trials). Center of mass (COM) excursion angles and mediolateral (ML) COM excursions were analyzed and used as outcome measures.

**Results:**

Group A showed higher COM excursion angles and ML-COM excursion on over-ground VR trials compared to NW trials (*p* < 0.05), while Group B only showed kinematic changes for the crowded VRE compared to NW trials during the treadmill walking protocol (*p* < 0.05). Post over-ground exposure, Group A showed greater COM excursion angle and ML-COM excursions on VR trials compared to NW trials during the treadmill walking protocol (*p* < 0.05). Post treadmill exposure, Group B only showed higher COM excursion angles for the snowy VRE compared to NW trials during the over-ground walking protocol (*p* < 0.01).

**Conclusion:**

Results showed that higher kinematic gait adjustments in response to VRE demands were observed during over-ground walking. Additionally, higher sensorimotor responses to VRE demands were observed when the VR protocol was first performed on the over-ground surface and followed by the treadmill walking condition (Group A) compared to the opposite (Group B).

## 1. Introduction

Gait adaptation is a notable ability of humans with important implications for meeting environmental demands [1, 2]. These adaptations are made possible by the sensorimotor integration of visual and proprioceptive information, which is essential to produce appropriate locomotor modifications for safe and successful interactions with both predicted and unpredicted environmental variables [3, 4]. Vision is primarily used in a feedforward manner to identify environmental variables, such as attributes of potential obstacles or surface irregularities [5–7], while proprioceptive input is critical for the maintenance and production of the initial plan based on the attributes identified from the visual information [8, 9].

A widely-used strategy to modify environmental feedback during motor tasks is the use of virtual reality environments (VREs) [10–13]. Virtual reality (VR) has been defined as the use of interactive simulations created with computer hardware and software to give users opportunities to be engaged with environments that appear and feel similar to the real world [10, 12]. Recent advancements in VR technology offer access to unexplored paradigms by providing a safe environment to analyze how humans react and adapt to various types of VREs and discordant sensorimotor stimulations [14, 15].

Numerous variables influence the level of immersion to VREs. The fidelity of a virtual environment, which means how similar the content of the virtual stimulation is to the real world [10, 12]; the users' ability to participate in and modify the virtual environment (level of interactivity); and technical factors such as objective properties of the display, navigation methods, and user interfaces can influence in the subjective feeling of presence or “being there” [16]. However, it has been described that some VR stimulations can produce negative effects in users, such as disorientation or nausea [17]. These effects are more likely to be produced by more immersive technologies which can interfere with the experience of “feeling present” [18]. Thus, for the efficient implementation of VR training protocols, there are two seemingly conflicting goals. The first one is related to the capability of one system to induce a proper immersion experience to the users. The second one is related to the cost of these devices and how to help others avoid costly or wasteful situations in which highly immersive systems are not necessary [19]. In this context, it has been reported that visualizations that are less complex, more regular, easier to understand, and with less immersive experience might perform as well as the more immersive ones [19]. Among these lines, Piccione et al. showed that ecological validity to VRE was more influenced by photorealistic environments provided by passive virtual immersion paradigms rather than polygon-modelled environments provided by active virtual immersion paradigms. This finding suggests that using recordings of real environments may contribute to a greater feeling of presence and that animated simulations may restrict the immersion experience [18].

VR-based gait training is a common therapeutic strategy for gait rehabilitation in populations with neurological diseases and older adults who experience mobility problems [20–25]. The addition of VREs to walking interventions provides an enriched environment for training by simulating conditions similar to what users experience during daily living [23]. Further, users find the incorporation of VR during tasks to be more interesting, thereby facilitating more time for practice with a higher number of repetitions [21, 23].

The use of VREs during treadmill walking is also becoming increasingly popular in the area of rehabilitation medicine [25–28]. Several studies have shown that VR-based treadmill training was beneficial for improving parameters such as symmetry and increased walking speed in populations with sensorimotor disabilities [25, 26]. Additionally, it has been described that ground reaction force (GRF) components during gait are similar between treadmill and over-ground gait, showing only some kinematic changes between those two surface conditions, thus demonstrating that both types of gait can be compared [29]. However, other studies reported that the addition of VREs to treadmill walking does not normalize comfortable walking speed to over-ground values and instead decreases stride length and increases step width variability [27, 28]. These findings are interpreted as a sign of a more cautious gait, potentially induced by instability due to the perceptual mismatch between optical flow and the walking speed imposed by treadmill devices [28–31].

The relevance of the integration of visual and sensorimotor feedback to gait and balance performance has been largely studied in the past years [5–8]. Peripheral feedback from the legs can modify locomotor activity throughout the central pattern generators (CPGs) in order to provide an appropriate movement according to external demands [32]. On the other hand, it has been well described that visual information is relevant in feedforward control of locomotion, and that optic flow should also be able to modify locomotor activity shown in terms of a modulation in three main components of locomotion: walking speed, stride length, and cadence [6, 33]. In this context, gait adjustment in response to VRE demands could be affected depending on the surface in which the VR protocol is implemented, which in turn could also interfere with the transference for the kinematic gait adaptation to VRE demands from one surface to other. Additionally, it remains unknown how variable practice (i.e., walking under different VREs) influences human locomotor behavior when participants are asked to adapt their walking pattern to novel context [34] (i.e., changes in the surface walking conditions).

Although it has been reported that gait training using VR promotes sensorimotor adaptations [15, 20, 22], the differences in motor responses to VRE demands while walking on different surface conditions, such as over-ground and treadmill, remain controversial. Additionally, the level of transfer of these sensorimotor responses to VRE demands from one surface condition to another remains unclear.

The first aim of this study was to examine if healthy young subjects could demonstrate immersion-induced kinematic responses, assessed by center of mass (COM) excursion angle and mediolateral (ML) COM excursion, to different passive photorealistic VREs during over-ground and treadmill walking conditions. The second aim of this study was to examine if the order in which the training surface conditions are provided affects the kinematic gait responses to VRE demands. We hypothesized that greater immersion-induced kinematic responses would be observed when the exposure to passive photorealistic VREs was performed on the over-ground surface condition compared to the treadmill condition. We also hypothesized that, due to the sensory conflict between somatosensory inputs and the visual information generated when the VR protocol was performed on the treadmill, participants who perform the VR protocol on the treadmill condition first, followed by the over-ground condition, would show less consistent kinematic gait adjustment responses to VRE demands than those who perform the VR protocol on the over-ground condition before the treadmill walking protocol.

## 2. Methods

### 2.1. Participants

Twenty healthy young participants (9 females and 11 males) took part in this study. Participants were separated into two groups (Group A (6 males and 4 females) and Group B (5 males and 5 females)). General demographic information is reported in Table 1. All the participants were prescreened and excluded if they had a self-reported history of any type of neurological or peripheral sensory disorder; musculoskeletal, visual, cardiopulmonary, and/or systemic disorders that could affect the locomotor-balance control system; any surgeries within last 6 months; and skin lesions that could not be protected appropriately. All participants provided written informed consent approved by the Institutional Review Board in the University of Illinois at Chicago.

### 2.2. Study Design

**Figure 1:**
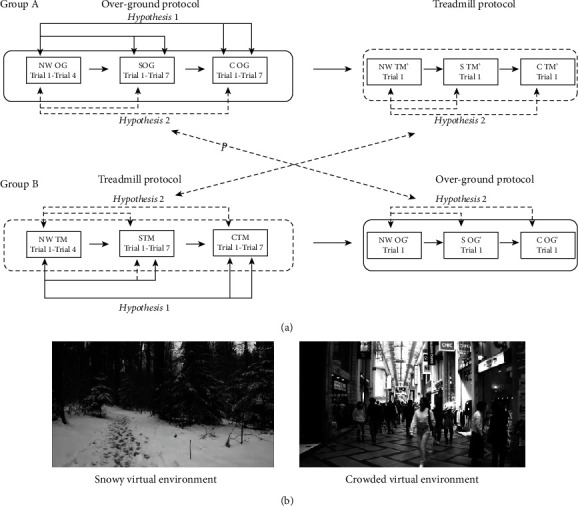
(a) Schematics of study design. Two groups (Group A and Group B) experienced VR protocol training over-ground and on a treadmill. Group A performed the VR over-ground protocol that included 4 natural walking (NW) trials, seven snowy (S) VRE trials, and seven crowded (C) VRE trials, followed by a VR treadmill protocol that included one NW trial, one S trial, and one C trial. Group B performed the VR treadmill protocol that included four NW trials, seven S VRE trials, and seven C VRE trials, followed by a VR over-ground protocol that included one NW trial, one S VRE trial, and one C VRE trial. Initial adaptation to VRE with respect to NW trials was compared between groups to test hypothesis 1. Initial kinematic changes in response to the first exposure to each VRE for each surface condition were compared between groups to test hypothesis 2. (b**)** Still-frame from the snowy and crowded virtual environment videos.

This study used an experimental design and included two VR protocols, the over-ground to treadmill VR protocol and the treadmill to over-ground VR protocol (Figure 1(a)). Each protocol included walking trials under two passive photorealistic VRE (snowy (S) and crowded (C)) conditions in addition to baseline natural walking (NW) trials (walking without a head-mounted display (HMD)). In the snowy VRE, participants were asked to walk in a snowy forest in which there was a path full of snow and ice. In the crowded VRE, participants were asked to walk down a crowded hallway among many people. However, in order to not provide any additional stimulus, any potential collision with the people included in this virtual scenario generated a change in the VRE configuration (Figure 1(b)). The choice of the virtual environments used in this protocol was associated to the facts that the risk of falling is higher when people are walking in the community and that the overall number of people presenting with fracture after a fall on snow and ice conditions remains more than double compared to other conditions [35]. Participants were assigned to either the over-ground to treadmill group (Group A) or the treadmill to over-ground group (Group B). Participants from Group A were asked to perform the VR over-ground protocol before performing the VR treadmill protocol. Conversely, Group B was asked to perform the VR treadmill protocol before the VR over-ground protocol. For the over-ground trials, participants walked at their self-selected speed on a 7-meter-walkway. For the treadmill trials, participants were asked to walk on a treadmill at 0.70 m/s for 12 seconds. In order to familiarize the participants with the experimental surface used in this protocol, they were asked to walk for 5 minutes on the treadmill at a speed of 0.7 m/s with and without the head-mounted display (HMD) before starting the VR treadmill protocol, and three times on the over-ground surface, with and without the HMD, before starting the over-ground VR protocol. All participants were instructed to navigate the environment they see as much as possible, and follow the virtual variables included in the scenarios in the current protocol (snowy and crowded VREs).

#### 2.2.1. Virtual Reality Over-Ground to Treadmill Protocol

The protocol started with four natural walking (NW1-NW4) trials as baseline. Participants then completed four trials in a snowy VRE (S1-S4) and four trials in a crowded VRE (C1-C4). Following these three blocks of trials, participants performed three trials for each VRE in a randomized order, thus receiving a total of seven trials each for the snowy (S1-S7) and crowded (C1-C7) VREs. After VRE exposure for over-ground walking, participants were asked to walk on a treadmill for four natural walking trials (NWTM1′-NWTM4′) followed by one exposure each to the snowy (STM′) and crowded (CTM′) VREs (Figure 1(a)).

#### 2.2.2. Virtual Reality Treadmill to Over-Ground Protocol

Participants were asked to walk on a treadmill where they performed four baseline natural walking (NWTM1-NWTM4) trials, four walking trials in a snowy VRE, and four trials in a crowded VRE followed by an additional three trials of each VRE in a randomized order (STM1-STM7 and CTM1-CTM7). Following the VRE exposure on the treadmill condition, participants were asked to walk on the over-ground walkway for four natural walking trials (NW1′-NW4′), followed by one exposure each to the snowy (S′) and crowded (C′) VREs (Figure 1(a)).

### 2.3. Experimental Setup

An eight-camera motion analysis system (Motion Analysis Corporation, Santa Rosa, CA) recorded data at 120 Hz from 28 reflective markers placed bilaterally on the upper (one marker on the wrist, one marker on the elbow, and one marker on the shoulder) and lower extremities (three markers were placed on the foot, one on the tibia, one marker on the knee, one marker on the thigh, and one marker on the hip), on the torso (one marker on the sacrum, one marker on the right scapula, and one marker on C7), on the head (one marker on each ear and one marker on the top of the head), and on the over-ground walkway (two markers). Marker displacement data was low-pass filtered at a marker-specific cut-off frequency (range 4.5-9 Hz determined through a residual analysis, Winter 2005) using fourth-order Butterworth filters.

The ActiveStep treadmill (Simbex, Lebanon, NH) was used during the VR treadmill protocol to control walking speed and trial duration. This treadmill device was wide enough with sufficient flat surface outside the treadmill belt. Additionally, if participants stepped on the surface outside the treadmill belt, we stopped the trial for safety reasons (this situation happened three times from a total of 240 trials performed on a treadmill). All participants wore running shoes and were connected to a full-body safety harness attached to a load cell by a pair of shock absorbing ropes which were attached to a friction ceiling-mounted track to protect them from injuries.

The VR videos for the snowy (snow-walking video downloaded from VaR's PRO Virtual Reality app) and crowded (shopping arcade (Osaka, Japan) video downloaded from VAR's Pro Virtual Reality app) conditions were played on a cell phone attached to a low-cost light-weight ProView head-mounted display (HMD) which showed the scene using two different images, one for the left eye and one for the right eye, allowing users to see the scenes at a resolution of 680 by 800 pixels using VaR's PRO Virtual Reality application. HMD have been shown to be safe and well accepted in young and older adults as well as efficient to deliver immersive VR interventions [36]. In the current protocol, the speeds of the optical flow provided for the VR system was not synchronized with the users' walking speed, and no additional stimulation and/or information other than the photorealistic video was delivered from the VR device.

### 2.4. Outcomes Variables

**Figure 2:**
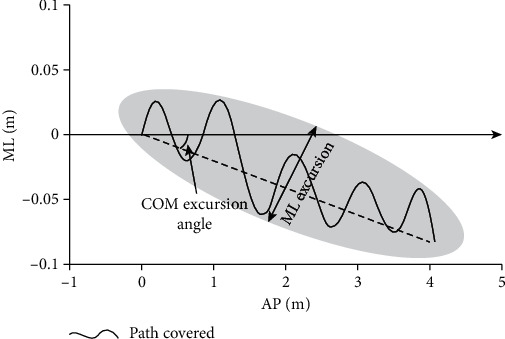
Graphical representation of the over-ground walkway describing how the kinematic outcome variables (COM excursion angle and ML-COM excursion) were measured in relation to the participant's walking path.

To measure motor behavior during the walking trials for each study condition, two kinematic outcome measures, using the center of mass (COM) as reference, were analyzed. Center of mass position was computed from the kinematic data using known gender-dependent segmental parameter information in a 13-segment representation of the body [37]. Center of mass velocity was obtained as the first numerical differentiation of the COM position. The first kinematic outcome measure was the excursion angle of the COM, which was defined as the deviation of the COM relative to the sagittal plane. The COM excursion angle was established to be the angle generated by the line connecting the starting and final position of the COM on the transverse plane and the projected line on the sagittal plane (Figure 2).

The second kinematic parameter analyzed was the mediolateral (ML) excursion of the COM relative to the walking direction, which was defined as the peak excursion of the COM perpendicular to the walking direction. The ML excursion of the COM was the minor radii of a 95% confidence ellipse area:

(1) $$\begin{array}{c} 95\%\text{confidence ellipse}={\left[F_{0.05\left[2,n-2\right]}\left(s_{\text{AP}}^{2}+s_{\text{ML}}^{2}-D\right)\right]}^{1/2}, \end{array}$$

where *F*_0.05[2, *n* − 2]_ is the *F* statistic at a 95% confidence level for a bivariate distribution with *n* data points. *s*_AP_^2^ and *s*_ML_^2^ are the standard deviations of the AP and ML time series, respectively, and

(2) $$\begin{array}{c} D={\left[\left(s_{\text{AP}}^{2}+s_{\text{ML}}^{2}\right)–4\left(s_{\text{AP}}^{2}s_{\text{ML}}^{2}-s_{\text{APML}}^{2}\right)\right]}^{1/2}, \end{array}$$

where *s*_APML_ is the covariance.

It has been well described that the motion of the COM itself is a constant target of neural control which could be used to describe gait and balance performance [38]. Additionally, relating the COM motion to the segmental kinematic perspective allows the understanding of adaptative mechanisms during gait [38]. In this context, both COM kinematic outcome measures reported in this study were considered as kinematic markers of the level of interaction of the participants with the VREs. Similarly, the analysis of the COM excursion angle and the COM ML excursion can reflect the kinematic response to VRE in both over-ground and treadmill conditions, assuming that in the treadmill condition the spatiotemporal parameters and walking direction were restricted for safety reasons and because of the dimensions of the treadmill.

As a complement to the kinematic outcome variables, walking speed was assessed for each group during the baseline natural walking trials on the over-ground condition and was calculated as the average speed of the COM during each trial.

### 2.5. Statistical Analysis

To test whether all data obtained from our outcome measures was normally distributed, a Shapiro-Wilk test was performed. Participant's baseline kinematic data (COM excursion angle and ML-COM excursion) during NW trials on the over-ground and treadmill conditions and their walking speeds during NW and NW′, S1 and STM1, and C1 and CTM1 trials were compared between groups using paired *t*-test analysis. The effect of surface condition on kinematic responses to VRE demands was examined by a 2 × 3 ANOVA with “group” (Group A and Group B) as the between factor and “trials” (NW, S1, and S7 for the snowy VRE and NW, C1, and C7 for the crowded VRE) as the repeated factor.

To test whether the order in which the training surface conditions were provided affected the sensorimotor responses to VRE demands, a 2 × 2 repeated measures ANOVA was performed individually for the snowy and crowded VREs. For this analysis, “group” served as the between factor and the first natural walking trial and the first virtual exposure trial for each surface condition served as repeated factors (e.g., NW and S1 for Group A were compared with NW′ and S1′ for Group B). Significant main effects and interactions were followed up with post hoc Bonferroni correction. To verify the magnitude of the changes after the intervention, the effect size (ES) was calculated based on partial eta squared (*η*_*p*_^2^) and Cohen's d. Effect size is classified as follows: small (0.0–0.20), medium (0.30–0.50), and large (0.50–0.80). All analyses were performed with SPSS 22 (IBM, Chicago, IL).

## 3. Results

Two groups each with ten participants, Group A (25.53 ± 1.45) and Group B (25.80 ± 1.22), completed the study protocol. No demographic differences were observed between groups (Table 1). With regard to baseline outcome measures, no differences between groups were observed for COM excursion angle or ML-COM excursion for the first natural walking trial on both over-ground and treadmill surface conditions (*p* > 0.05) (Table 2). Additionally, no between-group differences in baseline walking speed were observed for NW and NW′, S1 and STM1, or for C1 and CTM1 trials (*p* > 0.05) (Table 2).

### 3.1. Effect of Initial VRE Exposure on Gait Adaptation

**Figure 3:**
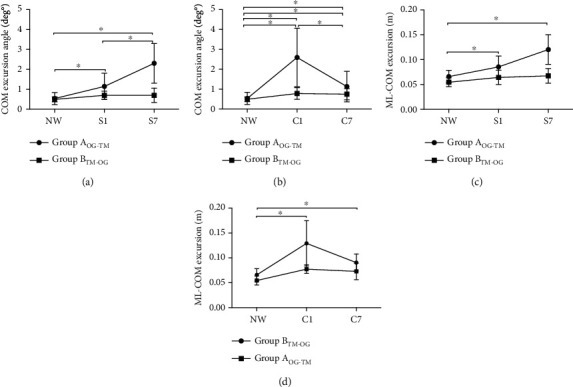
Between-group comparative analysis of the kinematic adjustments (COM excursion angle and ML-COM excursion) in response to the first exposures to the VREs. (a) Mean and SD of the COM excursion angle for the natural walking trial (NW), first snowy VRE trial (S1), and seventh snowy VRE trial (S7). (b) Mean and SD of the COM excursion angle for the first natural walking trial (NW), first crowded VRE trial (C1), and seventh crowded VRE (C7). (c) Mean and SD of the ML-COM excursion for the first natural walking trial (NW), first snowy VRE trial (S1), and seventh snowy VRE trial (S7). (d) Mean and SD of the ML-COM excursion for the first natural walking trial (NW), first crowded VRE trial (C1), and seventh crowded VRE trial (C7). Significant changes between trials (*p* < 0.05) are indicated by ∗.

The 2 × 3 ANOVA for COM excursion angle indicated a significant main effect of trial (VREs) (*F*(2, 17) = 22.2, *p* < 0.01, *η*_*p*_^2^ = 0.72) and a group∗trial interaction effect (*F*(2, 17) = 11.9, *p* < 0.01, *η*_*p*_^2^ = 0.58) for the snowy VRE (Figure 3(a)) and a significant main effect of trial (*F*(2, 17) = 12.5, *p* < 0.01, *η*_*p*_^2^ = 0.58) and a group∗trial interaction effect (*F*(2, 17) = 7.55, *p* < 0.01, *η*_*p*_^2^ = 0.47) for the crowded VRE (Figure 3(b)). Post hoc analysis demonstrated that Group A showed a higher COM excursion angle for S1 (first snowy VR trial) (*p* < 0.01; *d* = 0.59) and S7 (last snowy VR trial) (*p* < 0.01; *d* = 0.83) over-ground trials compared to baseline NW. In addition, the COM excursion angle for S7 was significantly higher than for S1 (*p* < 0.01; *d* = 0.76). No changes were observed between NWTM and STM1 (first snowy VR trial) and STM7 (last snowy VR trial) for Group B (*p* > 0.05). On the other hand, both groups showed higher COM excursion angles during crowded VRE trials compared to natural walking (*p* < 0.05), and the Group A COM excursion angle in C7 (last crowded VR trial) was significantly smaller than in C1 (first crowded VR trial) (*p* < 0.01; *d* = 0.75), although it continued to stay higher than NW (*p* < 0.05; *d* = 0.45).

Similarly, the 2 × 3 ANOVA for ML-COM excursion indicated a significant main effect of trial (VREs) (*F*(2, 17) = 17.5, *p* < 0.01, *η*_*p*_^2^ = 0.67) and a group∗trial interaction effect (*F*(2, 17) = 6.2, *p* < 0.01, *η*_*p*_^2^ = 0.42) for the snowy VRE (Figure 3(c)) and a significant main effect of trial (*F*(2, 17) = 21.5, *p* < 0.01, *η*_*p*_^2^ = 0.71) and a group∗trial interaction effect (*F*(2, 17) = 4.05, *p* < 0.01, *η*_*p*_^2^ = 0.32) for the crowded VRE (Figure 3(d)). For the snowy VRE, Group A showed higher ML-COM excursions during S1 (*p* < 0.01; *d* = 0.51) and S7 (*p* < 0.01; *d* = 0.79) compared to NW, and the ML-COM excursion for S7 was significantly higher than for S1 (*p* < 0.01; *d* = 0.68). Conversely, there were no changes for Group B between NWTM and STM1, between NWTM and STM7, and between STM1 and STM7 (*p* > 0.05). For the crowded VRE, Group A showed higher ML-COM excursions during C1 (*p* < 0.01; *d* = 0.73) and C7 (*p* < 0.01; *d* = 0.54) compared to NW, with the ML-COM excursion for C7 decreasing significantly compared to C1 (*p* < 0.01; *d* = 0.44), although they continued to stay higher than for NW (*p* < 0.05; *d* = 0.41). For Group B, no changes in ML-COM excursion were observed during CTM1 and CTM7 compared to NWTM (*p* > 0.05), and no differences in ML-COM excursion were observed between CTM1 and CTM7 (*p* > 0.05).

### 3.2. Effect of Surface Condition Exposure Order

**Figure 4:**
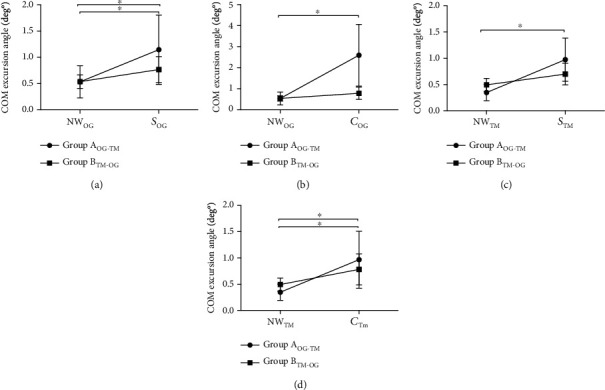
Comparative analysis of the COM excursion angle in response to the first exposure to the VRE for both over-ground and treadmill surface conditions. (a) Mean and SD of the COM excursion angle for the first natural walking (NW_OG_) trial and the first snowy (*S*_OG_) VRE trial during over-ground walking. (b) Mean and SD of the COM excursion angle during the first natural walking (NW_OG_) trial and the first crowded (*C*_OG_) VRE trial during over-ground walking. (c) Mean and SD of the COM excursion angle for the first natural walking (NW_TM_) trial and the first snowy (*S*_TM_) VRE trial during treadmill walking. (d) Mean and SD of the COM excursion angle for the first natural walking (NW_TM_) trial and the first crowded (*C*_TM_) VRE trial during treadmill walking. Significant changes between trials (*p* < 0.05) are indicated by ∗.

A 2 × 2 ANOVA to examine the effect of the order of surface condition exposure for the over-ground condition demonstrated a significant main effect of trial (*F*(1, 18) = 18.3, *p* < 0.01, *η*_*p*_^2^ = 1.01) for COM excursion angle, but not a significant group∗trial interaction (*F*(1, 18) = 3.02, *p* = 0.09) for the snowy VRE (Figure 4(a)). For the snowy VRE, Group A showed a greater COM excursion angle for S1 (their first exposure to the snowy VRE) compared to NW (NW vs. S1, *p* < 0.001; *d* = 0.76), and Group B showed a greater COM excursion angle for S1′ compared with NW′ during the over-ground protocol post treadmill exposure (NW′ vs. S1′, *p* < 0.01; *d* = 0.51). For the crowded VRE, a main effect of trial (*F*(1, 18) = 25.4, *p* < 0.01, *η*_*p*_^2^ = 0.58) and a group∗trial interaction (*F*(1, 18) = 16.2, *p* < 0.01, *η*_*p*_^2^ = 0.47) for COM excursion angle was observed for the over-ground condition (Figure 4(b)). However, unlike the increased COM excursion angle observed in Group A for the initial over-ground exposure to the crowded VRE (C1 > NW, *p* < 0.05; *d* = 0.49), Group B demonstrated no changes in COM excursion angle for *C*′ compared to NW′ (NW′ vs. *C*′, *p* > 0.05).

For COM excursion angle during the treadmill condition, there was a significant main effect of trial (*F*(1, 18) = 24.3, *p* < 0.01, *η*_*p*_^2^ = 0.57) and a group∗trial interaction (*F*(1, 18) = 10.6, *p* < 0.01, *η*_*p*_^2^ = 0.37) for the snowy VRE (Figure 4(c)), while for the crowded VRE there was a significant main effect of trial (*F*(1, 18) = 18.9, *p* < 0.01, *η*_*p*_^2^ = 0.51) but no group∗trial interaction effect (*F*(1, 18) = 0.6, *p* < 0.01) (Figure 4(d)). Group A demonstrated a significant increase in COM excursion angle during STM′ and CTM′ compared to NWTM′ (*p* < 0.01; *d* = 0.55), while Group B showed an increase in COM excursion angle during CTM1 compared to NWTM (*p* < 0.05; *d* = 0.43) but no difference for STM1 compared to NWTM (*p* > 0.05).

**Figure 5:**
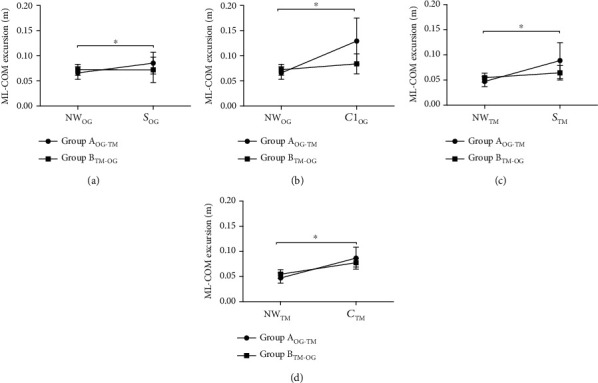
Comparative analysis of the ML-COM excursion in response to the first exposure to the VRE for both over-ground and treadmill surface conditions. (a) Mean and SD of the ML-COM excursion for the first natural walking (NW_OG_) trial and the first snowy (*S*_OG_) VRE trial during over-ground walking. (b) Mean and SD of the COM excursion angle for the first natural walking (NW_OG_) trial and the first crowded (*C*_OG_) VRE trial during over-ground walking. (c) Mean and SD of the ML-COM excursion for the first natural walking (NW_TM_) trial and the first snowy (*S*_TM_) VRE trial during treadmill walking. (d) Mean and SD of the ML-COM excursion for the first natural walking (NW_TM_) trial and the first crowded (*C*_TM_) VRE trial during treadmill walking. Significant changes between trials (*p* < 0.05) are indicated by ∗.

For ML-COM excursion during the over-ground condition, no main effect of trial (*F*(1, 18) = 3.5, *p* < 0.01) and no group∗trial interaction effect (*F*(1, 18) = 0.06, *p* > 0.05) was found for the snowy VRE (Figure 5(a)). However, a main effect of trial (*F*(1, 18) = 24.3, *p* < 0.01, *η*_*p*_^2^ = 0.57) and a group∗trial interaction effect (*F*(1, 18) = 11.9, *p* < 0.01, *η*_*p*_^2^ = 0.39) were found for the crowded VRE (Figure 5(b)). There was a significant increase in ML-COM excursion from NW to S1 (*p* < 0.05; *d* = 0.47) and from NW to C1 for Group A (*p* < 0.05; *d* = 0.49), but no changes in ML-COM excursion were observed from NW′ to S1′ and from NW′ to C1′ for Group B (*p* > 0.05).

For the treadmill surface condition, a main effect of trial (*F*(1, 18) = 12.7, *p* < 0.01, *η*_*p*_^2^ = 0.41) and a group∗trial interaction effect (*F*(1, 18) = 5.6, *p* < 0.01, *η*_*p*_^2^ = 0.23) were observed for the snowy VRE (Figure 5(c)). Similarly, a main effect of trial (*F*(1, 18) = 60.5, *p* < 0.01,*η*_*p*_^2^ = 0.77) and a group∗trial interaction effect (*F*(1, 18) = 5.8, *p* < 0.01, *η*_*p*_^2^ = 0.24) for the ML-COM excursion were observed for the crowded VRE (Figure 5(d)). For Group A, after initial exposure to the over-ground condition, the ML-COM excursion increased significantly from NWTM′ to STM′ (*p* < 0.01; *d* = 0.57) for the snowy VRE during the treadmill protocol. On the other hand, Group B showed no differences in ML-COM excursion for STM compared to NWTM (*p* > 0.05). Additionally, for the crowded VRE, Group A showed a significant increase in ML-COM excursion for CTM′ compared to NWTM′ (*p* < 0.05; *d* = 0.45), while Group B did not show any differences in ML-COM excursion for the first crowded VRE trial provided on the treadmill compared to natural walking (NWTM vs. CTM, *p* > 0.05).

## 4. Discussion

This study demonstrated that healthy young adults showed greater levels of kinematic gait adjustments in response to VRE demands during walking on an over-ground surface than during walking on a treadmill as assessed by COM excursion angles and ML-COM excursions. Additionally, more consistent overall immersion-induced kinematic responses to VRE demands were observed when the initial exposure to the VREs was performed on the more realistic over-ground surface compared to being first provided on the treadmill condition.

### 4.1. Kinematic Responses to VREs across Trials

Based on the study results, it was observed that participants from Group A significantly increased their COM excursion angles and ML-COM excursions in S1 and C1 trials compared to baseline (NW) and in S7 compared to S1 (Figures 3(a) and 3(c)); however, both COM excursion angle and ML-COM excursion decreased in C7 compared to C1 (Figures 3(b) and 3(d)). These kinematic adjustments were interpreted as a motor response according to the specific demands of each VRE, inferring that participants walked with greater caution in S1 (first snowy VR trial), but as trials progressed (S7), participants from Group A became more comfortable with the VRE demands, allowing them to walk more confidently and with a greater degree of freedom for COM displacements (Figures 3(a) and 3(c)).

In the crowded VRE, participants from Group A exhibited greater COM excursion angles and ML-COM excursions in the first trial (C1) compared to NW, which could be related to the participants' intention to avoid colliding with people immersed in the crowded VR video, which in turn produced an overcorrection during gait. Along these lines, it has been described that clearance of obstacles can be modified by the obstacles' characteristics. For instance, pedestrians modify their clearance when crossing an aperture formed by two people as opposed to poles [38] or when avoiding human-like avatars compared to inanimate objects immersed in VREs [39]. However, in the later trials (C7), participants began anticipating the VRE accurately, and thus only slight adjustments were needed to avoid collisions with the virtual people (Figures 3(b) and 3(d)).

On the other hand, when the first VRE trials were performed on the treadmill (Group B), the kinematic responses to VRE demands were less robust and participants preferred to remain stable instead of showing significant kinematic adjustments to enhance the immersion experience. Another alternative that could explain these results is that treadmill walking, by nature, generates less mediolateral movement than in over-ground walking; thus, it is possible that participants may have experienced the same levels of interactions with the VREs as in the over-ground condition but were using a constrained walking mode. Hence, either of the above postulations could explain the lack of differences observed in COM excursion angles or ML-COM excursions during snowy VRE treadmill trials (STM1-STM7) compared to the baseline treadmill trials (NWTM). Although, there were significant changes in COM excursion angles in CTM1 and CTM7 compared to NWTM, no differences in COM excursion angles were observed between CTM1 and CTM7 (Figure 3(b)).

### 4.2. Transference of Kinematic Adjustments in Response to VRE Demands between Surface Conditions

After the over-ground trials, participants from Group A performed the treadmill protocol in which COM excursion angles (Figures 4(a) and 4(c)) and ML-COM excursions (Figures 5(a) and 5(c)) were higher in STM and CTM compared to NWTM, which could reflect better walking confidence during the VR immersion experience because of the previous exposure to this VRE during the over-ground condition. Thus, despite the incongruence between the sensory information provided by the treadmill (mandatory speed of 0.70 m/s) and the inputs emerging from the VRE, participants from Group A were still able to maintain some kinematic adjustments made from the VR over-ground protocol during their performance on treadmill. Based on these results, we can infer that the kinematic adjustments in response to VRE demands made by participants from Group A during the over-ground protocol were strong enough to be conserved even when a mismatch between somatosensory and visual input was induced (VR trials on treadmill).

Participants from Group B, however, performed the over-ground protocol after the treadmill trials and only a slight increase in COM excursion angles was observed during the snowy (*S*′) VRE compared to NW′ (Figure 4(a)). Accordingly, it is possible that the incongruence between the somatosensory information provided by the established treadmill speed and the visual feedback provided by the VRE affected the sensorimotor responses to VRE demands of participants from Group B during their first exposure to the VRE (performed on treadmill), which in turn remained during the following over-ground protocol.

Previous studies reported that the gait kinematic changes in response to VRE demands are highly dependent on setup characteristics (i.e., VR domes or headset), the mode of walking (i.e., self-paced treadmill, fixed-speed treadmill, or over-ground walking), and the time given to adapt to the new environment [26, 31, 40, 41]. Our study confirms these previous findings, showing that the kinematic adjustments performed by the participants during VR trials were in response to the demand of each VRE, and also that these kinematic adjustments were more pronounced on the over-ground condition than on the treadmill condition. However, to our knowledge, this is the first study to report differences in gait kinematic responses to VRE demands based on the order in which the surface conditions were provided.

It is well known that vestibular, visual, and somatosensory systems contribute to the ability to maintain postural stability during gait [5, 6]. The protocol used in our study did not manipulate vestibular and somatosensory cues, allowing us to infer that VREs influence gait performance more at a central, as opposed to a peripheral, level of processing, and thus the kinematic adjustments observed during the VRE trials were in response to the VRE demands (visual inputs). Although somatosensory and vestibular inputs were not manipulated during the experiment, a conflict between the peripheral (somatosensory) and central (visual) sensory systems was induced during the VR treadmill protocol. The passive photorealistic VREs used in our protocol were depictions of walking in a snowy environment and in a crowded environment where participants should have been trying to avoid collisions with people, but they were also required to walk along a straight path on the treadmill (VR treadmill protocol). Thus, because of the differences between the optical flow provided by the VR system and the somatosensory information provided by the treadmill belt surface, the somatosensory and visual systems may have been providing a different reference frame for the control of gait. Further, the resolution of such conflict between the two sensory systems might be challenging considering the constant treadmill speed [33, 41, 42]. On the other hand, during the over-ground protocol, participants were receiving a more natural somatosensory stimulation from the surface and were not forced to walk at a constant speed, so they had the chance to adjust their spatiotemporal gait parameters according to the visual information received from the VREs. These differences between both VR protocols may explain the minor amount of kinematic adjustments in response to the VRE demands during the treadmill protocol compared to the over-ground protocol.

Our results demonstrated that the gait adjustments, generated as a result of young adults' interaction with different VREs, can be maintained even for conditions in which somatosensory information differs from the conditions where these gait adjustments were acquired. However, the level of the sensorimotor responses to different VRE demands was conditioned to the order in which the training surface conditions were provided. Thus, higher levels of kinematic responses to the VRE demands were observed when training was first provided on the over-ground surface compared to the protocol in which the VR protocol was first provided on the treadmill surface.

Additionally, our results show that to achieve improved levels of kinematic responses and interaction rates to VRE demands, the sensory information (visual, somatosensory, and vestibular) should be congruent during the acquisition period of the task. As was seen in this study, the inclusion of VR during treadmill walking could challenge the development of sensorimotor responses to VRE demands, which could potentially be prejudicial for patients in the early stage of the rehabilitation process, but, at the same time, could also be beneficial to challenge patients with lesser motor impairments or those in outpatient programs.

The results of the study should be interpreted considering its limitations. The study only had 20 participants and these participants were healthy young adults who are not most representative of the populations which could best benefit from this training paradigm. Further, the fact that the treadmill group walked at a preset constant speed, but the protocol did not enforce the over-ground group to do the same, could also be seen as a limitation because walking speed differences between groups could affect the outcome measures assessed in this study. However, there were no significant differences in walking speeds between the two groups during their self-selected natural walking trials on the over-ground condition, and no statistical differences were observed between groups during the initial VR trials for both snowy and crowded VREs performed on over-ground and on treadmill (Table 2). In addition, the HMD and software used to display the VREs in our protocol did not synchronize the optical flow with the walking speed which could significantly affect the users' immersive experience to the VREs. However, studies have shown that passive VR protocols that have included photorealistic VREs had better levels of immersion than active VR protocols [18, 19], which demonstrate that this kind of VR protocols are feasible and can potentially be used in other populations such as older adults and populations with neurological disorders. Finally, cybersickness was not assessed in the current protocol, which could be used as an additional outcome measure or exclusion criteria. However, none of the participants reported nausea or any other discomfort related to the exposure to VR. Future studies should include cybersickness in the design of VR protocols.

Future related research should examine gait adaptation abilities of different populations with sensorimotor disorders using training with VREs. Both the snowy and crowded VREs represent challenging scenarios in which there are higher incidence of falls. Potential new studies could use the information reported in this study to reproduce the current protocol in populations under high risk of falling, such as older adults and/or stroke populations. Future studies may also benefit from involving different VREs, including scenarios that better represent daily activities, as well as incorporating different VR devices which could improve the immersion experience.

## Figures and Tables

**Table 1:** Comparison of demographic characteristics between both groups in the study. Abbreviations: y = years; cm = centimeters; ns = not significant.

	Group A (*n* = 10)	Group B (*n* = 10)	*p* value
Age (y)	25.53 ± 1.45	25.80 ± 1.22	0.90 (ns)
Gender			
Male, *n* (%)	6 (60%)	5 (50%)	
Female, *n* (%)	4 (40%)	5 (50%)	
Height (cm)	169.1 ± 1.01	163 ± 3.43	0.13 (ns)

**Table 2:** Between-group comparisons of baseline COM excursion angles and ML-COM excursions during the first natural walking trial (NW) for over-ground and treadmill (NWTM) walking, and between-group comparisons of walking speed during NW, NW′, and during the first exposure to VRE (snowy and crowded VRE).

Baseline assessments	Group A (mean ± SD)	Group B (mean ± SD)	*p* value
COM excursion angle for the first over-ground NW trial (degrees)	0.534 ± 0.08	0.538 ± 0.04	0.96 (ns)
COM excursion angle for the first treadmill NWTM trial (degrees)	0.347 ± 0.04	0.494 ± 0.03	0.06 (ns)
ML-COM excursion for the first over-ground NW trial (m)	0.065 ± 0.003	0.072 ± 0.003	0.21 (ns)
ML-COM excursion for the first treadmill NWTM trial (m)	0.047 ± 0.002	0.054 ± 0.002	0.072 (ns)
Walking speed for the first over-ground trial (m/s) (NW vs. NW′)	0.91 ± 0.136	0.97 ± 0.122	0.316 (ns)
Walking speed for the first snowy VRE trial (m/s) (S1 vs. STM1)	0.73 ± 0.16	0.70 (predetermined)	0.55 (ns)
Walking speed for the first crowded VRE trial (m/s) (C1vs. CTM1)	0.73 ± 0.17	0.70 (predetermined)	0.53(ns)

Abbreviations: COM = center of mass; NW = natural walking; NWTM = natural walking on treadmill; NW′ = natural walking after previous exposure to VR protocol on treadmill; S1 = first snowy VRE trial on over-ground; STM1 = first snowy VRE trial on treadmill; C1 = first crowded VRE trial on over-ground; CTM1 = first crowded VRE trial on treadmill; ML = mediolateral; m = meters; and ns = not significant.

## Data Availability

The data used to support the findings of this study are available from the corresponding author upon request.
